# The Use of Rheological and Tribological Techniques for Texture Assessment of Ambient Yoghurt

**DOI:** 10.3390/foods15030440

**Published:** 2026-01-26

**Authors:** Shuli Hu, Hui Li, Hongliang Li, Hairan Ma, Yajun Fei, Xiuying Wu, Wenbin Zhu, Jianshe Chen, Shuanghong Li

**Affiliations:** 1 Laboratory of Food Oral Processing, School of Food Science and Biotechnology, Zhejiang Gongshang University, Hangzhou 310018, China; shulihu37@163.com (S.H.); zhuwenbin_2023@163.com (W.Z.); 2 Inner Mongolia Mengniu Dairy (Group) Co., Ltd., Hohhot 011500, China; lihui6@mengniu.cn (H.L.); lihongliang@mengniu.cn (H.L.); mahairan@mengniu.cn (H.M.); feiyajun@mengniu.cn (Y.F.); wuxiuying@mengniu.cn (X.W.)

**Keywords:** ambient yoghurt, graininess, slipperiness, sensory evaluation, rheology, tribology

## Abstract

**Background**: Ambient yoghurt, also known as room-temperature yoghurt, has gained increasing attention due to its convenience in distribution and consumption without needing cold storage. To ensure extended shelf life, ambient yoghurt normally undergoes an additional heat treatment during manufacturing, the post-fermentation sterilisation process (typically at 65–85 °C), which may induce the formation of fine particle aggregates and result in undesirable textural attributes, particularly graininess. Assessing textural attributes of such products remains a challenge. **Methods**: By mimicking the oral behaviour of ambient yoghurt, this study uses rheological as well as tribological techniques for objective assessment of the textural sensations of slipperiness and graininess. Various experimental conditions, including the amount of saliva incorporation, sliding speed, and ball-contact and plate-contact lubrication, were examined, and results were analysed against perceived texture by panellists. **Main findings**: The results indicate that viscosity changes are closely associated with perceived slipperiness under the tested conditions. The friction coefficient obtained from a plate-contact tribometer shows a positive correlation with the sensation of graininess (Pearson’s r was 0.74, *p* < 0.05, N = 8). It was also observed that a 20% saliva incorporation showed the closest agreement with sensory perception, although this observation should be interpreted cautiously due to the limited sample size. **Implications**: Results obtained from this work indicate the feasibility of using rheology and tribology techniques for texture prediction in ambient yoghurt. The findings are exploratory in nature, and further studies with larger sample sets are required to validate the proposed approach. The methodology presented here may serve as a reference framework for investigating texture perception in other dairy systems.

## 1. Introduction

Ambient yoghurt is a nutrient-rich dairy product that has attracted increasing attention [1]. Unlike its traditional version, the manufacturing of ambient yoghurt usually involves three separate heat treatments, i.e., pasteurisation during the pretreatment of raw milk, heat treatment during the homogenisation process, and pasteurisation after fermentation [2]. The specific temperature–time profiles of these treatments depend on several factors, such as heating method, milk type, viscosity after fermentation, pH, and the addition of thickeners or stabilisers [3]. Improper heat treatment conditions, such as overly fast temperature increases or overly high fermentation temperatures, may induce protein precipitation and the formation of fine particle aggregates [4]. The presence of such aggregates is known to result in texture defects and undesirable sensory attributes.

Previous studies have demonstrated that sensory graininess in yoghurt is closely associated with the presence of small particles dispersed within the continuous phase [5]. Very early on, Kokini et al. [6] suggested that the presence of fine particles would have both rheological and tribological implications, and a well-known model was developed to explain their contributions to the perceived slipperiness and smoothness of dairy products. The sensation and perception of fine particles during oral processing can be even more complicated. During oral processing, yoghurt is stirred by the tongue, leading to a rising temperature, decreasing viscosity, and progressive incorporation of saliva, all of which can substantially alter its oral behaviour and sensory perception [7]. Consequently, instrumental assessment and prediction of sensory attributes in yoghurt systems remain challenging.

Rheology has always been seen as a main tool for sensory characterisation of yoghurt and many other dairy products [8]. Flow behaviour, apparent viscosity, and viscoelasticity have been widely used in the literature as feasible parameters for objective assessment of the sensory attributes of such products [9]. These rheological properties primarily describe the bulk deformation and flow behaviour of yoghurt, which are particularly relevant during the early stages of oral processing, when the product undergoes compression, shearing, and mixing by the tongue and palate.

In recent years, tribology has also attracted much attention from food science researchers for its potential role during oral processing and, of course, sensory perception [10]. It is speculated that bulk deformation and flow could play a determining role during the early stage of oral processing, but oral tribological behaviour could gradually become dominant during the later stage of oral processing as a result of a thin layer of fluid between the tongue and hard palate [11]. Tribological measurements therefore provide information on interfacial lubrication and frictional interactions, which are closely related to sensory attributes, such as slipperiness and smoothness, perceived at later stages of oral processing.

However, despite the extensive tribology research reported in the literature, establishing a close link between lubrication measurement and sensory perception remains a major challenge [12]. Lack of a reliable measurement technique could be one of the reasons behind this. Currently, a large number of tribological devices have been reported [13], but most of these devices are based on ball-on-plate or point-contact designs, through which friction is measured by moving (either rolling and/or sliding) a ball against a flat substrate [14]. While such a design is applicable for fluid systems with no suspended particles, it could potentially be a mismatch for colloidal food systems due to the fact that such a design may fail to entrap particles. This could be particularly true for many dairy systems, such as yoghurt, which contain particle aggregates across a wide range of sizes.

To address these challenges, the present study aims to explore an integrated rheological–tribological approach to investigate texture perception in ambient yoghurt, with a particular focus on slipperiness and graininess. By employing complementary tribological configurations using both ball–plate and plate–plate contacts, this study seeks to better capture the influence of particle aggregates under lubrication conditions relevant to oral processing.

Specifically, a set of nine ambient yoghurts with differing viscosities was examined using sensory evaluation, rheological measurements, and tribological testing, both in the absence and presence of saliva. The relationships between particle size, particle hardness, system viscosity, and sensory perception were analysed to provide mechanistic insights into texture perception. The proposed approach is evaluated in an exploratory manner using model yoghurt samples, with the aim of assessing its potential applicability for texture characterisation rather than establishing definitive predictive models.

## 2. Materials and Methods

### 2.1. Material

#### 2.1.1. Yoghurt Preparation

The viscosity of yoghurt plays a dominant role in oral processing, as well as in rheological and tribological measurements [3,4]. For this study, nine commercially available yoghurt samples were selected from the market and categorised into three groups based on their apparent shear viscosity (measured at 25 °C and 50 s^−1^): the low-viscosity group (3 samples): A, B, C, with a viscosity between 0.48 and 0.51 Pa·s; the medium-viscosity group (3 samples): D, E, F, with a viscosity between 0.53 and 0.70 Pa·s; and the high-viscosity group (3 samples): G, H, I, with a viscosity between 0.72 and 0.89 Pa·s. For convenience, all samples were labelled alphabetically.

Their main compositions, as provided by the manufacturer and labelled on the packaging, are listed in Supplementary Table S1. It should be noted that these commercial yoghurt samples differed in formulation, including fat content, protein content, sugar composition, and stabilisers, which may influence rheological, tribological, and sensory properties. Therefore, compositional variability is acknowledged as an inherent limitation of the present study.

Although the grouping was based on apparent viscosity at a representative shear rate (50 s^−1^), additional rheological parameters, including flow curves and viscoelastic properties, were measured and analysed to provide a more comprehensive characterisation of the non-Newtonian behaviour of the yoghurt samples.

All samples were stored and used according to the manufacturer’s instructions, without further alteration, and prior to their best-before date.

#### 2.1.2. Preparation of Artificial Saliva

Artificial saliva was prepared for experimental use. The ingredients of the artificial saliva were based on the method of Karthik et al. [15], consisting of mainly electrolytes, mucin, and amylase (Supplementary Table S2). This composition was selected to approximate key physicochemical characteristics of human saliva relevant to lubrication and enzymatic activity during oral processing of dairy matrices.

After the components were weighed and dissolved, 1 mol/L hydrochloric acid solution was used to adjust its pH to 6.8, and 500 mL was prepared each time and used fresh.

For tribological and rheological measurements, yoghurt–saliva mixtures were prepared at defined incorporation levels. The selected saliva addition ratios were intended to represent simplified in vitro dilution conditions during oral processing rather than exact physiological saliva uptake and were used to comparatively evaluate the influence of saliva incorporation on lubrication behaviour and sensory perception.

#### 2.1.3. Preparation of Model Yoghurt Samples with Different Thermal Treatment

Model yoghurt samples were prepared to further validate the feasibility of the tribology method for texture characterisation. These samples were made at the Inner Mongolia Mengniu Dairy (Group) Co., Ltd. Laboratory (Hohhot, China), and the production methods and detailed parameters are as follows [16]. The preparation and thermal treatment were conducted using three independently prepared batches to ensure experimental reproducibility. Same-batch samples were divided into three groups, canned in tubes, and pasteurised for 10 min at 65, 75, and 85 °C. Thermal treatment at different temperatures led to varying levels of particle aggregation, affecting the graininess of the yoghurt. Samples at 65 °C had low graininess, those at 75 °C had moderate graininess, with tiny particles, and samples at 85 °C had high graininess, with obvious particle presence. After thermal treatment, the samples were cooled and stored in a refrigerator at 4 °C. They were brought to room temperature 3 h before the test.

### 2.2. Sensory Evaluation

Sensory characteristics of consistency, slipperiness, and graininess were assessed by a sensory panel using Quantitative Descriptive Analysis [17]. The panel consisted of 12 panellists (6 males and 6 females, aged between 22 and 25 years old) who were regularly trained on and experienced in sensory analysis. Given the exploratory nature of the present study, this panel size was considered appropriate for comparative texture assessment.

Prior to assessment, panellists were asked not to consume any food or non-water beverages for 2 h prior to the experiments. All panellists gave informed, written consent to participate in the experiment. The experiment followed all ethical requirements set by the Experimental Ethics Committee of Zhejiang Gongshang University (No. 2023133937). Panels were non-smokers with no known smell or taste deficiencies and no reported oral or other health problems in the past six months.

During training sessions, panellists were asked to identify and define oral attributes. Definitions and methods of assessment are given in Table 1. Numerical scoring was employed for the sensory evaluation of yoghurt. Specifically, sensory attributes were assessed by a sensory panel, assigning scores ranging between 0 and 10, where 0 indicates the absence of the evaluated attribute and 10 represents an extremely high intensity. Reference samples were used during training to align the panel’s understanding of scale endpoints and improve scoring consistency.

#### The Ambient Yoghurt Graininess Sensation Focus Group

Yoghurt samples were evaluated at room temperature and were presented to each assessor in a random order. During assessment, the panellists were asked to scoop up a spoonful of yoghurt (about 4.0 ± 1.5 g), slowly transfer it into the mouth, gently press and move the tongue and sense for 15 to 20 s, and then swallow. After that, panellists were asked to rinse their mouth with 30 mL of water three times.

After assessing the three samples in each group, the panellists were asked to rank the graininess of all samples on a scale of 0–10 (10 being the strongest graininess, close to that of a soda cracker, and 0 being the weakest graininess, indicating no particles detected, with the reference sample being butter). Panellists were also allowed to provide qualitative comments on graininess perception.

The temporal evolution of graininess intensity was characterised using a time–intensity (TI) method implemented in Compusense software (Compusense Cloud, Version 8.8.6766.17069, Compusense Inc., Guelph, ON, Canada). Intensity ratings were continuously recorded during oral processing, and the resulting TI curves were used for subsequent comparative analysis.

### 2.3. Rheology Tests

Rheological measurements were carried out using a Discovery HR-2 rotary rheometer (TA Instruments, Leatherhead, UK). A conical plate clamp geometry (diameter 40 mm, angle 2.017°, operation gap: 55 μm) was used for the measurement over a shear rate ranging from 0.01 to 1000 s^−1^. The acquisition time of each data point was 30 s, and a total of 26 data points were collected across 5 orders of magnitude of shear rates. The three replicates refer to technical replicates, in which measurements were repeated on independently loaded aliquots from the same sample batch, and thus primarily reflect instrumental and measurement variability rather than batch-to-batch variation. All measurements/data are presented as the mean of three replicates. Apparent shear viscosity (η_s_), often simplified as shear viscosity, as a function of shear rate, was recorded.

### 2.4. Tribology Tests

Tribology tests were conducted using an established technique based on the Soft Texture Analyzer Tribometer (STAT) based on a Texture Analyzer TA.XTplus (Stable Micro Systems, Surrey, UK). The applicability and performance of this tribological setup for food-related lubrication studies have been reported previously [18]. The system comprises a texture analyser equipped with a customised sample chamber connected to a water circulation unit for temperature control. All experimental operations were controlled via the Texture Analyzer platform, and data acquisition was performed using Exponent software (Version 6.1; Stable Micro Systems, Surrey, UK).

During tribological testing, normal force, friction force, sliding distance, speed, and time were continuously recorded. The friction coefficient was calculated as the ratio of the measured friction force to the applied normal load. To minimise transient effects, the average friction force was determined from the steady-state region of the force–time curve during the sliding process. The identification of the steady-state region was based on the stabilisation of the friction signal after the initial running-in period.

This tribological configuration was employed to investigate lubrication behaviour under conditions relevant to oral processing, while acknowledging that the measurements represent simplified in vitro approximations of in-mouth lubrication phenomena.

### 2.5. Confocal Laser Scanning Microscope (CLSM) Observations

The microstructural properties of the yoghurt samples were measured by laser confocal microscopy (Olympus FLUOVIEW FV3000, Olympus Corporation, Tokyo, Japan). The protein excitation light was selected at 640 nm, while the fat excitation light was selected at 561 nm.

Before experiment, the fat and protein in the yoghurt samples were dyed with either Nile red or Fast green to effectively distinguish protein and fat [19]. Protein was stained by dissolving 1 mg of Fast green in 1 mL of dimethyl sulfoxide (DMSO) solvent, of which 50 μL was taken and mixed up with 950 μL of solvent dilution (DMSO) to obtain 0.05 mg/mL of protein stain. Fat was stained by dissolving 1 mg of Nile red in 1 mL of dimethyl sulfoxide (DMSO) solvent, of which 50 μL was taken and mixed up with 950 μL solvent dilution (DMSO) to obtain 0.05 mg/mL fat stain. Stained samples were carefully spread on a glass slide and covered with a cover sheet for microscope observation.

### 2.6. Particle Size Measurements

The particle size of protein aggregates in yoghurt was determined by Malvern Mastersizer 3000 (Malvern Instruments Ltd., Malver, UK). The yoghurt was gently dispersed into the sample tank with temperature controlled at 28 °C. Mean particle size was given as the volume/surface average or Sauter diameter D_4,3_. The refractive index (RI) was set to 1.5 and the absorption was set at 0.001 for measurements; these optical parameters were selected based on commonly reported values for dairy fat–protein aggregates in the literature and are widely used for particle size analysis of yoghurt and similar colloidal dairy systems [20].

### 2.7. Statistical Analysis

IBM SPSS (Version 26.0; IBM Corp., Armonk, NY, USA) Statistics was used for variance analysis, and Shapiro–Wilk (S-W) was used for data processing to test the normal distribution. All data were expressed as means and standard deviations under at least three repetitions. Differences among samples were evaluated using one-way analysis of variance (ANOVA), and statistical significance was defined at *p* < 0.05.

Rheological flow behaviour was characterised by fitting shear stress–shear rate data to the power-law model. Curve fitting was conducted using Prism 9.0 (GraphPad Software, San Diego, CA, USA) to obtain the flow behaviour index (n) and consistency coefficient (K). R Studio (Version 4.2.0; R Foundation for Statistical Computing, Vienna, Austria) was used for data visualisation and for constructing response surface plots to illustrate the relationships between selected instrumental variables and perceived slipperiness.

Correlations between instrumental parameters (rheological and tribological measurements) and sensory attributes were evaluated using Pearson correlation analysis. Due to the limited sample size, correlation results were interpreted in an exploratory manner.

## 3. Results and Discussion

### 3.1. Particle Size and Microstructure Characterisation of Yoghurt Samples

There is no doubt that the sensory attributes of ambient yoghurt are closely related to size, hardness, and concentration of particle aggregates in yoghurt [21]. In order to have a better understanding of the yoghurt samples, the particle size distribution and microstructure of all ambient yoghurt samples were examined using Mastersizer and CLSM techniques, respectively.

**Figure 1:**
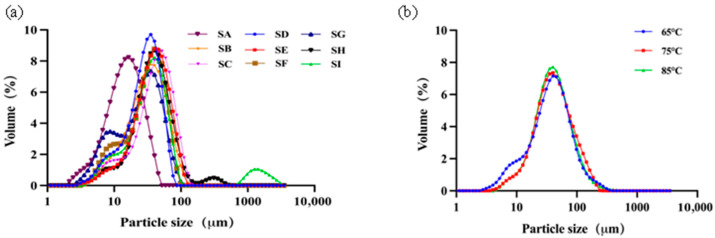
Particle size distribution of yoghurts and model yoghurt samples, the labels SA–SI represent Sample A–I (**a**), and model yoghurt samples treated at different temperatures (**b**).

Particle size distributions are given in Figure 1a for all yoghurt samples and their mean particle sizes were calculated and are given in Table 2. It appears that all yoghurt samples have a wide range of size distribution, mainly between 10 and 100 μm, either unimodal or bimodal. This description is based on qualitative inspection of the distribution curves rather than on formal quantitative modality analysis or fitting criteria.

The refractive index of oil/fat is in the range of 1.45–1.47, which is almost the same as the value used for the proteins (1.5). Observation by CLSM revealed that the fat and protein in yoghurt form aggregates; therefore, the particle sizes observed here are inferred to represent those fat–protein aggregates. This interpretation is supported by microscopic observation but does not constitute a quantitative confirmation, as no co-localisation coefficients or image-based statistical analyses were applied. The mean particle size D_4,3_ was between 17.3 and 51.5 μm for all yoghurt samples; Sample A had the smallest mean particle size, with its D_4,3_ of only 17.3 μm, while Sample G had the largest mean particle size, with its D_4,3_ of 51.5 μm. Specifically, the particle size distributions of Samples H and I appeared to be very different from others with clearly identifiable peaks on the higher end of particle size, larger than 100 μm or even 1000 μm. No statistical modality classification was applied, and the presence of large-sized peaks is therefore discussed descriptively. It can be assured that the presence of such large particles will, of course, affect oral sensation.

Figure 1b and Table 3 show the particle size distribution of model yoghurt samples. These model systems were obtained by undergoing different thermal treatment temperatures (Section 2.1.3). One can notice that thermal treatment at different temperatures has a limited effect on the mean particle size.

**Figure 2:**
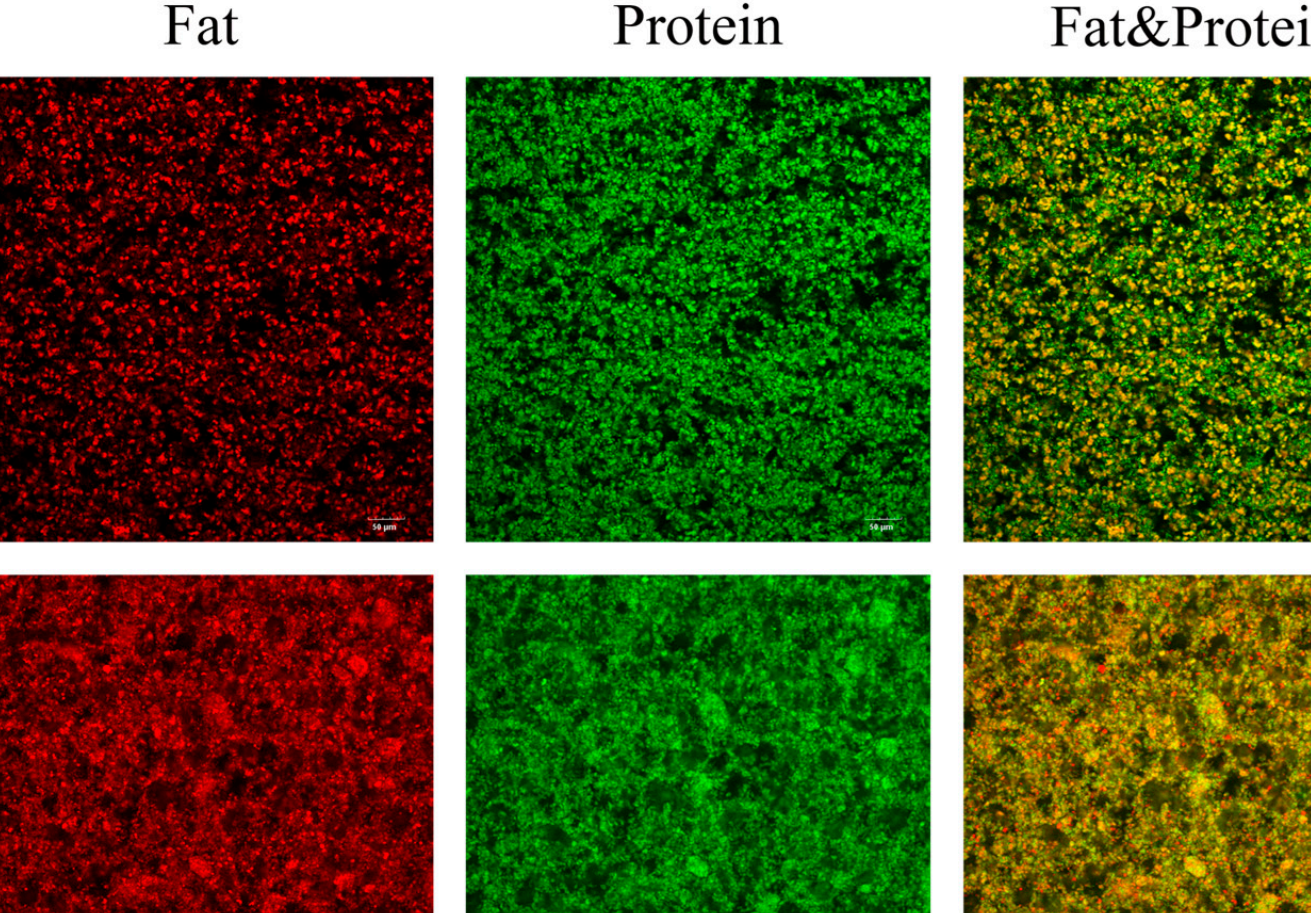
Distribution of fat and protein in representative yoghurt under CLSM (20×). Protein is shown in green (Fast Green staining), and fat is shown in red (Nile Red staining). Sample A represents low viscosity, higher slipperiness, and lower graininess, while Sample H denotes a sample with higher viscosity, lower slipperiness, and higher graininess.

Microstructural observations have been conducted for all yoghurt samples using the confocal technique. The protein and fat in yoghurt intertwine to form aggregates. Aggregations were clearly evident but to a greatly different extent among all yoghurt samples. Figure 2 shows the most representative cases of yoghurt microstructure, where Sample A represents a case of smooth microstructure, and Sample H represents a highly aggregated microstructure. The latter case exhibits a more porous and open microstructure, characterised by a significantly larger particle concentration.

### 3.2. Analysis of Sensory Attributes

Smooth sensation is one of the most appealing textural features for yoghurt and many other dairy products [22]. However, due to extensive thermal treatments during the manufacturing process and, as a result, the formation of excessive aggregated particles, ambient yoghurt suffers the obvious textural defect of grainy sensation [23]. This grainy sensation is also often perceived as gritty, chalky, powdery, or sandy [24]. Sensory analysis clearly indicates that all nine yoghurt samples were perceived as grainy, with Sample G being the most grainy and Sample A being the least (Table 4).

**Figure 3:**
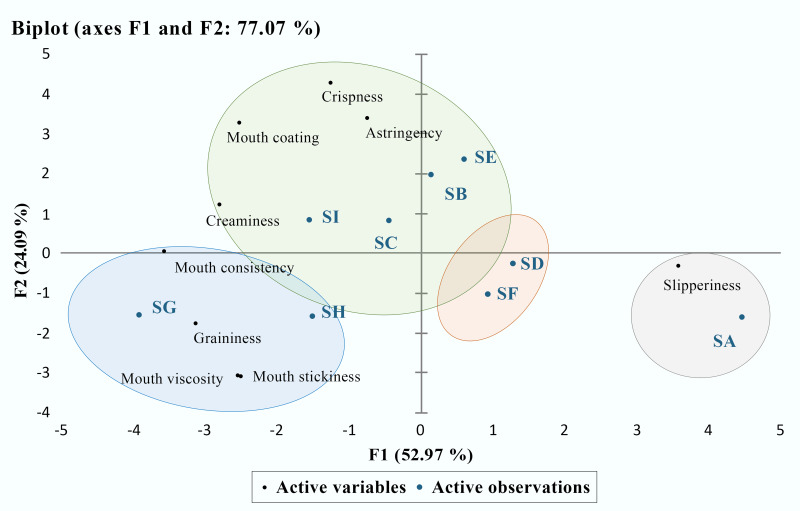
Principal Component Analysis of sensory perception of ambient yoghurt samples. Note: Colored ellipses are used as a visual aid to highlight groups of samples with similar sensory characteristics and do not represent statistical confidence regions.

Principal Component Analysis shows that yoghurt samples can roughly be divided into four groups (Figure 3). From the PCA biplot, graininess and slipperiness are positioned in opposite regions of the score–loading space, indicating that these two sensory attributes represent distinct and contrasting texture perception mechanisms. Samples located on the negative side of F1 and lower F2 values are closely associated with graininess-related attributes, including graininess, mouth viscosity, and mouth stickiness. This grouping corresponds to yoghurt samples characterised by a larger particle size and a higher degree of particle aggregation, suggesting that bulk structural heterogeneity plays a dominant role in the perception of graininess.

In contrast, samples located on the positive side of F1 are strongly associated with slipperiness. These samples are positioned far from the graininess-related loadings, indicating a sensory profile dominated by smooth oral motion and reduced particulate interference during oral processing. This sensory behaviour is consistent with samples exhibiting smaller effective particle structures and lubrication-dominated oral perception, where interfacial effects rather than bulk resistance govern mouthfeel.

Sensory consistency appears to be rather consistent with graininess sensation. Sample G has the highest sensation of oral consistency, while Sample A has the lowest intensity of that. However, the sensation of slipperiness is almost opposite to the sensation of graininess and oral consistency. The highest amount of slipperiness was observed for Sample A, and the lowest amount of slipperiness was observed for Sample G. Further correlation analysis revealed close relationships among three sensory attributes. The correlation analysis of sensory attributes showed that consistency was significantly positively correlated with graininess (Pearson’s r was 0.8648, *p* < 0.05, N = 9), and oral consistency was significantly negatively correlated with slipperiness (Pearson’s r was −0.9923, *p* < 0.05, N = 9).

**Figure 4:**
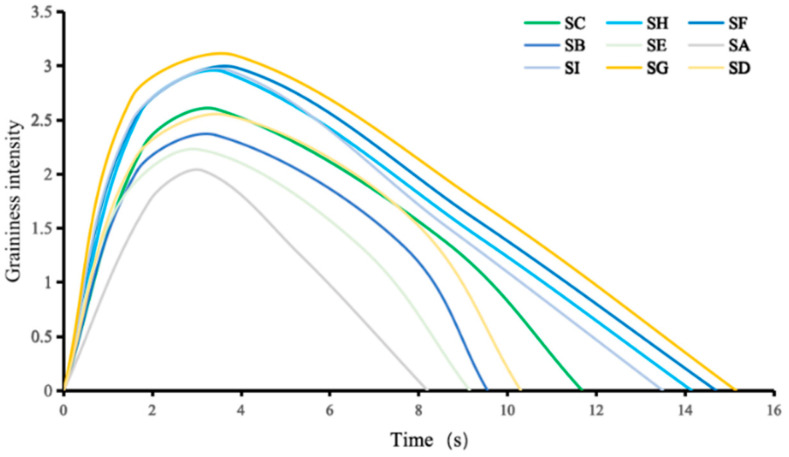
The curve of time–intensity of graininess. The intensity of the graininess is rated from 0 to 10. Timing starts from the moment of entry and stops when the graininess disappears.

Time–intensity analysis for all yoghurt samples reveals the dynamic change in the sensation during the eating process. Perceived intensity results are plotted as a function of oral processing time (Figure 4). One can see that the sensory graininess follows a more or less very similar pattern for all nine samples, i.e., a rapid increase at the early stage of oral processing, quickly reaching a maximum, followed by a unidirectional decrease until it is swallowed. The strongest graininess sensation was perceived between 3.1 and 3.9 s, and it disappeared between 8.2 and 15.2 s.

Subtle differences are still clearly observable among different samples, in particular, the maximum intensity and the lasting length of sensation. TI curves in Figure 4 suggest that perception of a textural attribute occurs quickly after ingestion, where the perceived intensity shows an initial rapid linear increase. Towards the later part of oral processing, oral shear and also the incorporation of saliva lead to particle disintegration or make such particles less perceivable.

The variation in the duration of perceived graininess in different types of yoghurt samples was found to be correlated with viscosity. Based on this observation, the following mechanistic interpretation is proposed. High-viscosity yoghurt flows more slowly in the mouth, maintaining prolonged contact with the tongue and oral mucosa. This extended contact time allows particulate matter or powdery components to linger on the tongue surface, thereby increasing the duration of the grainy sensation.

In contrast, low-viscosity yoghurt flows more readily, allowing its components to disperse more rapidly, resulting in a shorter grainy sensation. After swallowing, high-viscosity yoghurt is more likely to leave residues on the tongue, pharynx, and other areas of the oral cavity, which may contain fine particles, further prolonging the grainy sensation. In contrast, low-viscosity yoghurt typically clears from the mouth more quickly after swallowing, thus shortening the duration of the grainy sensation.

### 3.3. Saliva Incorporation During Oral Processing of Yoghurt

Saliva is an essential component of oral processing and has been widely studied in relation to eating behaviour and sensory perception due to its interactions with food matrices and its unique multilayer microstructure and functional properties [19,25,26,27]. It is generally accepted that sensory perception during consumption arises not only from the food itself but also from the evolving mixture of food and saliva. Consequently, understanding yoghurt–saliva interactions is important for interpreting texture perception during oral processing.

As indicated by the TI curves in Figure 4, high-viscosity systems tend to remain in the mouth for a longer duration. Yoghurt with a higher viscosity generally requires more mechanical processing inside the oral cavity, such as shearing, mixing, and tongue movement, in order to facilitate swallowing [28]. The increased surface contact area and extended duration between a viscous yoghurt and oral mucosa, combined with its reduced flowability, provide greater stimulation to the oral cavity [29]. However, it should be noted that these interpretations are based on in vitro measurements and sensory observations rather than direct physiological measurements.

Based on these observations, defined proportions of artificial saliva (20% and 50%) were incorporated into yoghurt samples in subsequent experiments to simulate simplified in vitro dilution conditions during oral processing. The levels of saliva addition were selected to comparatively examine the influence of saliva incorporation on lubrication behaviour and sensory perception, rather than to precisely replicate in vivo saliva uptake. These levels were proven to be appropriate by recent work on the sensory prediction of yoghurt products [30]. However, the results should still be interpreted as indicative of relative trends rather than absolute physiological responses.

### 3.4. Rheology

#### 3.4.1. Flow Behaviour and Perceived Consistency

**Figure 5:**
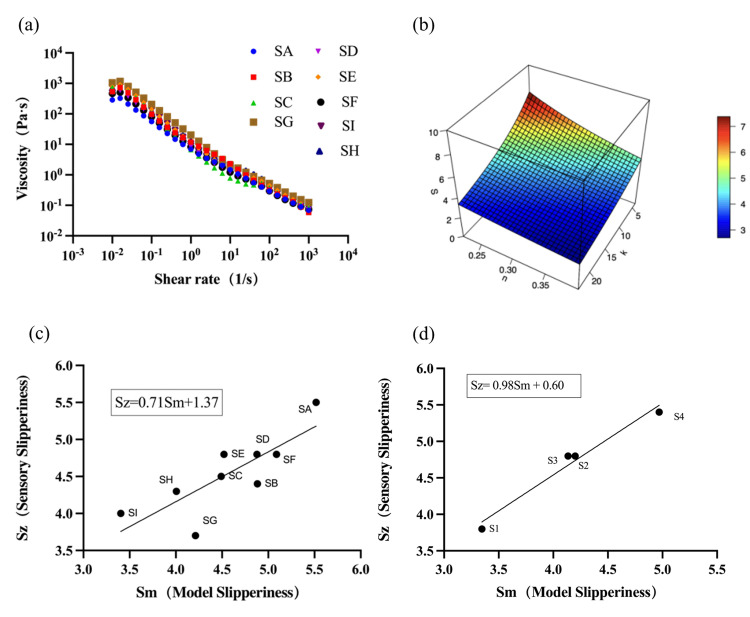
Rheology approach to sensory slipperiness of yoghurt. The curve of shear viscosity variation with shear rate at 25 °C (**a**). The 3D-fitted surface (**b**), the three axes are the sample’s n value, K value from rheology, and the sensory slipperiness scores. Correlation between sensory slipperiness, Sz, and predicted slipperiness, Sm, for nine commercial products (**c**) and validated products (**d**).

Flow behaviour tests were conducted for all yoghurt samples at 25 °C and results are shown in Figure 5a. It is no surprise that all yoghurt samples exhibited shear thinning, indicating increasing disassociation of particle aggregates under an increased shear rate. This result agrees with observations in the literature about yoghurt and many other soft solid colloidal systems [8]. While the monotonous viscosity decreases appeared to be very similar, differences were clearly evident among the nine samples. Take the apparent viscosity at 50 s^−1^ as an example; a strong correlation exists between the apparent viscosity measured at this shear rate and the oral consistency in the sensory test (Pearson’s r was 0.7270, *p* < 0.05, N = 9).

#### 3.4.2. A Prediction Model for the Slipperiness Sensation of Yoghurt

Slipperiness is a typical sensory attribute for fluid and soft solid foods. The sensory definition of slipperiness can vary slightly but largely reflects its dominating contribution from viscous flow and surface movement. Seo et al. [31] defined slipperiness as the “Degree of slide of the food bolus over the mucosal surface of the oral cavity and pharynx.” There have been many efforts to establish physical links for the sensory perception of slipperiness. The most classical analysis probably comes from Kokini et al. [6]. They proposed that sensory slipperiness is related to viscous force and friction force and suggested that slipperiness should be inversely proportional to the sum of viscous force and friction force, as shown below in Equation (1).

(1) $$\left(Slipperiness\right)\propto \frac{1}{\left[viscousforce+frictionforce1\right]}$$

Kokini’s model seems to be highly plausible and has been frequently referred toin the literature [32]. However, this model does not clearly distinguish the relative importance of the contributions from the viscous force and the friction force, but instead sets the two at an equal level. Even though Kokini et al. set viscous force and friction force as independent factors, as a matter of fact, friction force is also highly dependent on the viscosity of the lubricant [33]. Therefore, we intend to believe that viscous force is the dominating and most important contributing factor for sensory slipperiness and that it is possible for rheology data alone to predict the sensory perception of slipperiness.

Based on this consideration, the consistency index ‘*K*’ and flow behaviour index ‘*n*’ were obtained from the flow curves (Figure 5a). These two parameters are widely used rheological parameters in characterising the flow behaviour of non-Newtonian fluids. Together with the perceived intensity of slipperiness (*S*), a three-dimensional plot could be produced (Figure 5b). A correlation model between sensory slipperiness and the two indexes from the flow behaviour tests was then obtained:

(2) S=1/(aK+bn).

Further analysis based on the data shown in Figure 5b gave values of 0.008 and 0.451, respectively, for constants ‘a’ and ‘b’ for the above empirical model. Figure 5c plots the sensory slipperiness, *S*_z_, and the predicted slipperiness, *S*_m_, based on Equation (2), and we obtained a good correlation between the two (*R*^2^ = 0.6533, *p* < 0.05, N = 9). Given the limited sample size and the empirical nature of the model, this relationship should be interpreted as indicative rather than predictive.

To further examine the robustness of this empirical relationship, four additional commercial yoghurt samples (S1–S4) were evaluated using the same rheological measurements and sensory assessment procedures. Rheological parameters were used to estimate slipperiness based on Equation (2), and the estimated values were compared with sensory scores (Figure 5d). A high correlation was observed (*R*^2^ = 0.9709, *p* < 0.05, N = 4); however, this result should be interpreted with caution due to the small validation dataset and the absence of independent cross-validation. These findings suggest that rheological flow behaviour may provide useful insights into slipperiness perception, while highlighting the need for further validation using larger and more diverse sample sets.

### 3.5. Tribology

In order to establish a feasible tribological technique for objective measurement of texture perception of ambient yoghurt, two very different modes of tribological tests were adopted in this work: a ball-on-plate tribopair setup and a plate-against-plate tribopair setup. The former is capable of offering both sliding and rolling lubrication and has been widely used in the literature [34]. However, a major drawback of this setup is its limitation to dispersed systems due to the failed entrapment of particles during lubrication tests, therefore producing misleading results. A tribometer with a plate-against-plate tribopair was recently developed by our research group, and its feasibility and reliability have been tested [18]. Despite this experimental setup being able to operate on sliding friction only, it is appropriate and sufficient for oral lubrication, where the tongue moving against the hard palate basically involves one substrate surface (the tongue) sliding against another substrate (the hard palate and other oral surfaces). Most importantly, a plate-against-plate setup is fully capable of trapping suspended particles, offering a great advantage for tribological study of food suspension systems.

#### 3.5.1. Ball-on-Plate Tribological Tests

A ball-on-plate tribometer refers to a tribometer where a ball probe is moving (rolling or sliding) against a flat substrate. Due to the fact that high-viscosity systems easily to form thicker films between the probe and the supporting substrate surface (Polydimethylsiloxane (PDMS) in this case), these films effectively separate the surfaces, reducing direct contact and friction, and thereby lowering the coefficient of friction [35]. For high-viscosity samples, the stability of the lubricant film improves, enhancing the separation effect between friction surfaces and resulting in better lubrication performance [36].

At a low surface load (0.0535 N), the coefficient of friction for various samples increased with increasing sliding speed. This behaviour corresponds to the hydrodynamic region of the Stribeck curve [37], where a large gap between the spherical probe and the PDMS plate allows for a yoghurt film thick enough to support the entire load and minimise friction. Consequently, the lubrication characteristics depend highly on the properties of the yoghurt film. In particular, the apparent viscosity of the yoghurt has a high impact on the friction. High-viscosity systems exhibit lower friction results and better lubrication performance due to their good capability to form a sufficiently thick lubricating film, while low-viscosity yoghurt systems show higher friction results and poorer lubrication due to the fluid film being less capable to support the load. These measurement results align well with the fundamental principles of friction measurement [18].

To mitigate the lubrication scenarios of oral processing, nine yoghurt samples were mixed with artificial saliva at a 1:1 ratio. Following mixing, the samples were categorised into three groups based on their apparent viscosity: low-viscosity, medium-viscosity, and high-viscosity groups. Sample A, with an excessively low viscosity, was already in an easily flowable state prior to mixing, resulting in even lower viscosity post-mixing, while other yoghurt samples were in a viscous state. Sample E, originally classified in the medium-viscosity group, became part of the low-viscosity group after mixing, likely due to partial disintegration of particulates within Sample E, which significantly reduced its viscosity upon dilution.

**Figure 6:**
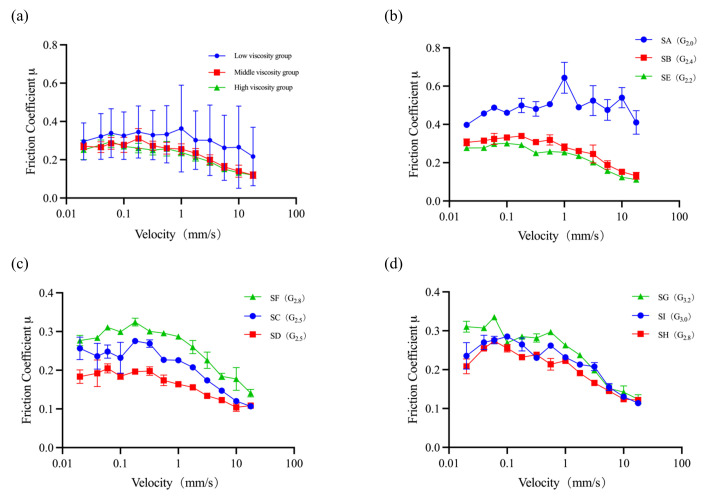
Tribological results of yoghurt with saliva measured using a ball-on-plate tribopair. The friction coefficient is plotted as a function of sliding speed: (**a**) Average friction coefficient of three groups after 1:1 mixing with artificial saliva; (**b**) yoghurt sample of low apparent viscosity; (**c**) yoghurt samples of medium apparent viscosity; (**d**) yoghurt samples of high apparent viscosity. The annotations in parentheses indicate the graininess scores. For example, SA (G_2.0_) means the graininess score of Sample A is 2.2.

The results obtained from ball-on-plate measurements are presented in Figure 6, where the friction coefficient is plotted against the sliding speed. While the friction coefficient shows some differences among yoghurt samples, it appears that this distinction is only at the category level (Figure 6a) for the high-viscosity, medium-viscosity, and low-viscosity groups. The three sample groups with varying apparent viscosities are likely situated in the boundary and mixed regimes of the Stribeck curve; viscosity continues to exert a dominant influence on the coefficient of friction (Figure 6a).

Within each group of similar apparent viscosity, the differences among yoghurt samples were noticeable though not very robust (Figure 6b–d). Figure 6b illustrates that the coefficient of friction for Sample A is significantly higher than that of the other samples. Among the remaining samples, those with higher graininess scores during sensory evaluation exhibited larger coefficients of friction, while those with lower graininess scores appeared to have smaller friction coefficients.

#### 3.5.2. Plate-Contact Tribological Tests

**Figure 7:**
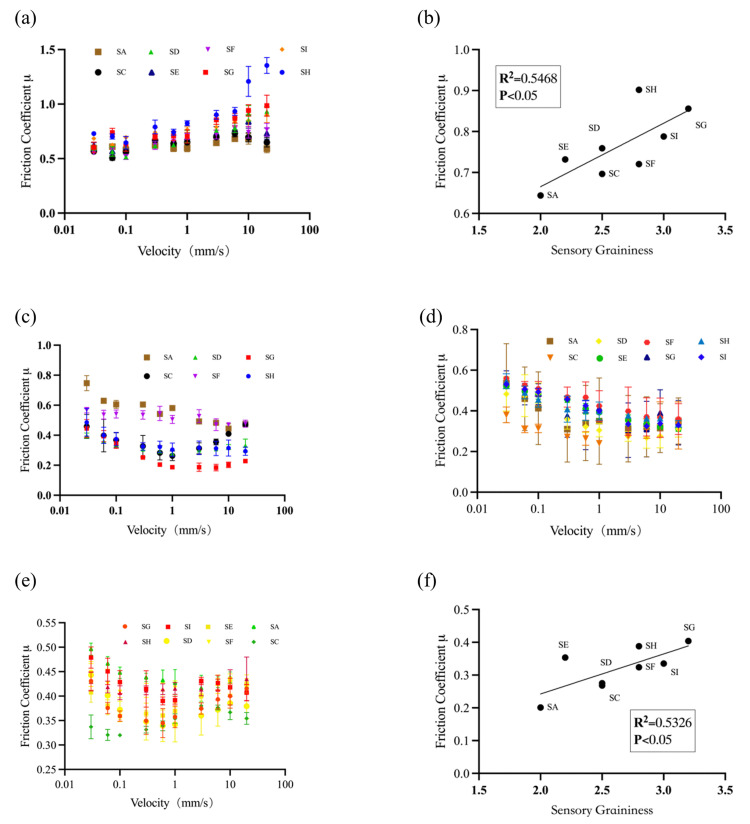
Tribology results of yoghurt and its saliva mixture obtained with plate-contact tribopair setup. Load of 0.0535 N without saliva addition (**a**); the corresponding regression analysis (**b**). Load of 0.4 N without saliva (**c**). Load of 0.4 N with 20% saliva (**d**). Load of 40 g with 50% saliva (**e**), the corresponding regression analysis at (**f**).

A ball-on-plate tribopair is, in nature, an instance of point-contact friction. A major concern of such a setup is its capability to entrap particles suspended within a lubricant system. To overcome this drawback, a plate-against-plate tribopair was developed, in which a ball probe was replaced by a plate probe [18]. A low surface load (0.0535 N) was first applied to the test, and the results are shown in Figure 7a. The friction coefficient shows a gradual increase with the increase in sliding speed, a case strongly suggesting a hydrodynamic region within a Stribeck curve, characterised by a fully developed lubricant film that separates the surfaces completely.

This contact configuration better mimics the oral processing of yoghurt, where a significant quantity of yoghurt is present in the mouth, between the tongue and hard palate and other oral surfaces. It seems that differences in the friction coefficient were minor at low sliding speeds. The differences became larger with the increase in sliding speed. Using the friction coefficient at 3 mm/s, one can see clear differences between yoghurt samples. At this sliding speed, a positive association was observed between friction coefficient and sensory graininess (*R*^2^ = 0.5468, *p* < 0.05, N = 8; Figure 7b). While this relationship indicates that plate-against-plate measurements may be sensitive to differences in particle-related texture attributes, the observed correlation should be interpreted cautiously due to the limited sample size.

Samples containing 20 % and 50 % saliva were tested, and the results are presented in Figure 7d,e. It seems that the friction curves for both cases were within the mixed and hydrodynamic regimes of the Stribeck curve. Additionally, the friction coefficient measured at a sliding velocity of 3 mm/s and 20 % saliva concentration showed a correlation with sensory perception of graininess (*R*^2^ = 0.5326, *p* < 0.05, N = 8) (Figure 7f). In contrast, no significant correlation was observed at 50% saliva incorporation (R^2^ = 0.3057, *p* > 0.1, N = 8). These results suggest that excessive saliva dilution may reduce the sensitivity of tribological measurements to particle-related sensory attributes. It should be emphasised that saliva incorporation levels in this study were selected for comparative in vitro evaluation rather than as precise representations of in vivo conditions.

The 20 % saliva incorporation in tribology tests of ambient yoghurt for prediction of oral texture sensation seems to agree very well with a separate study, in which the same ratio of saliva incorporation was found to give the best texture prediction for low-temperature yoghurts [30].

#### 3.5.3. Tribology Test of Model Yoghurt Samples

**Figure 8:**
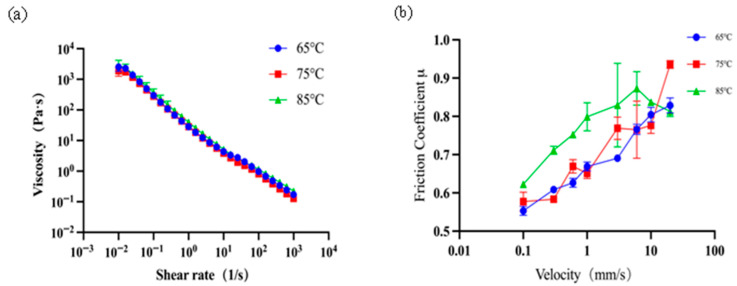
Rheological and tribological results of different yoghurt samples prepared under different thermal treatments. The flow behaviour of these samples was measured at 25 °C (**a**); lubrication behaviour was obtained by plate-contact tribopair (**b**).

To further verify the effectiveness of tribology analysis based on two different surface contacts, model systems prepared with different thermal treatments were examined. For validation purposes, yoghurt samples produced in the same batch, but which had undergone three different thermal treatment temperatures (65, 75, and 85 °C), were specifically prepared. Rheological analysis shows that the flow behaviour was very close (Figure 8a); particle size distributions also did not show big differences (see Figure 1b). However, sensory analysis showed a severe increase in graininess perception for the yoghurt sample treated at 85 °C.

The tribological tests produced very interesting results. Lubrication measurements results obtained from a ball-on-plate tribopair failed to give a clear distinction between these samples. However, results from the plate-contact test (Figure 8b) show that the friction curves at 75 °C and 65 °C are similar, but significantly lower than that at 85 °C. These results offer strong instrumental evidence and support to the analysis of graininess, suggesting that a plate-contact tribopair serves better for sensory prediction of yoghurt, at least in the case of graininess, where particle entrapment is essential. We believe that for particle-containing colloidal or suspension systems, where particle entrapping becomes an issue of concern, a plate-on-plate tribopair should provide a more suitable technique for instrumental lubrication characterisation.

## 4. Conclusions

In this work, sensory slipperiness and graininess were investigated with the aim of exploring their instrumental characterisation using rheological and tribological approaches.

Altogether, nine commercial samples were selected, and their microstructure and particle size distribution were observed. Sensory attributes were analysed by a trained panel. The flow behaviour of these yoghurt samples with or without saliva addition were characterised using a rheometer. It was found that the apparent shear viscosity measured at 50 s^−1^ was significantly positively correlated with the oral consistency (Pearson’s r was 0.7270, *p* < 0.05, N = 9). The empirical relationship between rheological flow parameters and slipperiness perception was further explored, suggesting that flow behaviour may provide useful insights into the sensation of slipperiness under the tested conditions. For instrumental characterisation of sensory graininess, tribological measurements were conducted for yoghurt samples using two different approaches: a ball-on-plate tribometer (point-contact) and a plate-on-plate tribometer (plate-contact). It was found that the plate-contact tribopair performs much better in distinguishing yoghurt samples where particle presence drastically impacts oral sensation. The friction coefficient showed a positive correlation with the sensation of graininess (Pearson’s r was 0.74, *p* < 0.05, N = 8). It was also observed that a 20% saliva incorporation gave the closest prediction to sensation perception. These observations should be interpreted cautiously given the limited sample size and the simplified in vitro nature of the measurements.

Overall, the findings suggest that sensory slipperiness and graininess are governed by different underlying mechanisms. Slipperiness appears to be primarily related to bulk flow behaviour, while graininess is more closely associated with lubrication behaviour, influenced by the presence of aggregated protein particles. This study highlights the potential of combining rheological and tribological measurements to gain complementary insights into texture perception of ambient yoghurt, while emphasising the need for further validation using larger datasets and physiologically relevant conditions.

## Figures and Tables

**Table 1:** Sensory attributes and evaluation methods.

Sensory Properties	Definition	Evaluation Method
Slipperiness	The degree of ease with which dairy products flow over the surface of the tongue is typically associated with aspects such as consistency and fineness.	Place a spoonful of the yoghurt on the front of the tongue, keeping the roof of the mouth clear, so the product can move back and forth across the tongue’s surface.
Graininess	The quantity of fine particles perceived on the surface of the tongue.	Place the yoghurt in the mouth and feel the particles squeezed on the roof of the mouth and tongue.
Mouth-consistency	The degree of thinness of a sample as perceived by stimulation of tactile receptors.	Spoon the yoghurt in the mouth and feel the its consistency.

**Table 2:** Mean particle size of yoghurt samples.

	Sample	SA	SB	SC	SD	SE	SF	SG	SH	SI
Size (mm)										
D_[3,4]_		17.3 ± 0.3	38.6 ± 3.2	48.3 ± 2.2	32.4 ± 1.5	43.9 ± 1.5	36.4 ± 1.1	51.5 ± 4.9	30.6 ± 0.6	37.5 ± 1.1
D_[2,3]_		11.9 ± 0.1	24.4 ± 1.4	28.6 ± 1.1	21.6 ± 0.8	28.6 ± 0.9	22.4 ± 0.5	29.0 ± 0.3	16.8 ± 0.3	25.9 ± 2.9
Dx (10)		6.3 ± 0.1	12.0 ± 0.6	12.9 ± 0.6	10.4 ± 0.2	15.4 ± 0.4	9.7 ± 0.1	15.6 ± 0.3	7.2 ± 0.1	11.4 ± 0.8
Dx (50)		15.5 ± 0.3	34.0 ± 2.6	44.8 ± 2.1	31.1 ± 1.4	40.3 ± 1.3	34.6 ± 1.2	40.3 ± 0.7	27.5 ± 0.5	37.6 ± 3.4
Dx (90)		31.2 ± 0.6	71.6 ± 6.6	86.8 ± 4.2	55.6 ± 3.1	77.8 ± 2.9	65.3 ± 1.9	85.0 ± 2.4	59.2 ± 1.1	85.4 ± 2.3

**Table 3:** Mean particle size parameters of the model yoghurt samples.

	T (°C)	65 °C	75 °C	85 °C
Size (mm)				
D_[3,4]_		50.7 ± 2.3	54.4 ± 0.9	53.1 ± 1.9
D_[2,3]_		25.7 ± 0.4	32.5 ± 0.2	32.1 ± 0.4
Dx (10)		11.4 ± 0.1	17 ± 0.1	16.9 ± 0.1
Dx (50)		40.1 ± 1.2	43 ± 0.3	41.8 ± 0.7
Dx (90)		97.7 ± 5.8	109.0 ± 3.1	99.9 ± 4.8

**Table 4:** Sensory attributes score from panel.

Analysis	SA	SB	SC	SD	SE	SF	SG	SH	SI	*p* Value
Mouth consistency	3.4	4.5	4.4	4.0	4.0	4.1	5.3	4.7	5.1	<0.0001 ***
Grade	F	D	D	E	E	E	A	C	B	
Graininess	2.0	2.4	2.5	2.5	2.2	2.8	3.2	2.8	3.0	<0.0001 ***
Grade	G	E	D	D	F	C	A	C	B	
Slipperiness	5.5	4.4	4.5	4.8	4.8	4.8	3.7	4.3	4.0	<0.0001 ***
Grade	A	CD	C	B	B	B	F	D	E	

Note: *** indicates statistical significance at *p* < 0.001.

## Data Availability

The original contributions presented in this study are included in the article/Supplementary Materials. Further inquiries can be directed to the corresponding authors.

## Supplementary Materials

The following supporting information can be downloaded at https://www.mdpi.com/article/10.3390/foods15030440/s1: Table S1. Details of yoghurt samples, samples number, the main components, the manufacture. Table S2. Components of artificial saliva, in addition to ionic ingredient, mucin and α-amylase have also been incorporated.
