# Supplementary material for: The Use of Rheological and Tribological Techniques for Texture Assessment of Ambient Yoghurt
Source: Foods. 2026 Jan 26;15(3):440. doi: 10.3390/foods15030440 (PMC12896520; doi:10.3390/foods15030440)
Supplement: Supplementary file 1 [file foods-15-00440-s001.zip › foods-4087725-supplementary.pdf]

Supplementary tables

Table S1. Details of yoghurt samples, samples number, the main components, the manufacture.

Table S2. Components of artificial saliva, in addition to ionic ingredient, mucin and α-amylase have also been incorporated.

`

Table S1. Details and components of yogurt samples.

Sample	Main compositions	Manufacture
Sample A	milk, white sugar, whey protein powder concentrate, hydroxypropyl distarch phosphate, pectin, AGAR, cream, fermented fungi	Xinjiang Garden Dairy Co., LTD
Sample B	milk, white sugar, whey protein powder, hydroxypropyl distarch phosphate, pectin, AGAR, fermentation fungi, food flavor	New Hope Dairy Co. LTD
Sample C	milk, white sugar, whey protein powder, acetylated distarch phosphate, pectin, AGAR, monodiglyceride diacetyltartrate, fermentation fungi, food flavor	Junlebao Dairy Co., LTD
Sample D	milk, white sugar, whey protein powder, hydroxypropyl distarch phosphate, pectin, AGAR, fermentation fungi	Inner Mongolia Mengniu Dairy Co., LTD
Sample E	milk, white sugar, concentrated whey protein powder, concentrated milk protein, evaporated milk, Jufen, hydroxypropyl distarch phosphate, pectin, AGAR, fermentation fungi, food flavor	Shandong Sanyuan Dairy Co., LTD
Sample F	milk, white sugar, whey protein powder, hydroxypropyl distarch phosphate, AGAR, sodium alginate, pectin, food flavor, fermentation fungi	Shanghai Bright Dairy&Food Co., LTD
Sample G	milk, white sugar, whey protein powder, cheese, hydroxypropyl distarch phosphate, polyglucose, gelatin, pectin, Gellan gum, AGAR, fermentation fungi	Inner Mongolia Mengniu Dairy Co., LTD
Sample H	milk, white sugar, whey protein powder, acetylated distarch phosphate, pectin, Gellan gum, AGAR, monodiglyceride diacetyltartrate, fermentation fungi, food flavor	Inner Mongolia Yili Industrial Dairy Co., LTD
Sample I	milk, white sugar, whey protein powder, hydroxypropyl distarch phosphate, pectin, AGAR, fermentation fungi, food flavor	Hangzhou adopted a cow Biotechnology Co., LTD

`

Table S2. Components of artificial saliva.

**Chemicals**	**Concentration (g/L)**	**Grade**	**Manufacturer**
Sodium chloride	0.111	AR	Shanghai Qiangshun Chemical Reagent Co., LTD
Potassium chloride	1.492	AR	Chengdu Colon Chemical Co., LTD
Sodium bicarbonate	3.948	AR	Chengdu Colon Chemical Co., LTD
Calcium chloride	0.278	AR	Merck Chemical (Shanghai) Co., LTD
Magnesium chloride hexahydrate	0.096	AR	Chengdu Colon Chemical Co., LTD
Mucin (porcine gastric Π type)	1.500	AR	Sigma Aldrich Chemical (Shanghai) Co., LTD
α-amylase (4000U/g 1G)	2.000	AR	Jiaxing Maya Reagent Co., LTD
